# Recent advances in extracellular vesicles for therapeutic cargo delivery

**DOI:** 10.1038/s12276-024-01201-6

**Published:** 2024-04-01

**Authors:** Hyo In Kim, Jinbong Park, Yin Zhu, Xiaoyun Wang, Yohan Han, Duo Zhang

**Affiliations:** 1 Department of Surgery, Beth Israel Deaconess Medical Center, Harvard Medical School, Boston, MA 02215 USA; 2 Department of Pharmacology, College of Korean Medicine, Kyung Hee University, Seoul, 02447 Republic of Korea; 3 Clinical and Experimental Therapeutics, College of Pharmacy, University of Georgia and Charlie Norwood VA Medical Center, Augusta, GA 30912 USA; 4 Department of Microbiology, Wonkwang University School of Medicine, Iksan, 54538 Republic of Korea; 5 Sarcopenia Total Solution Center, Wonkwang University, Iksan, 54538 Republic of Korea; 6 Department of Medicine, Medical College of Georgia, Augusta University, Augusta, GA 30912 USA

**Keywords:** Nanoparticles, Drug delivery

## Abstract

Exosomes, which are nanosized vesicles secreted by cells, are attracting increasing interest in the field of biomedical research due to their unique properties, including biocompatibility, cargo loading capacity, and deep tissue penetration. They serve as natural signaling agents in intercellular communication, and their inherent ability to carry proteins, lipids, and nucleic acids endows them with remarkable therapeutic potential. Thus, exosomes can be exploited for diverse therapeutic applications, including chemotherapy, gene therapy, and photothermal therapy. Moreover, their capacity for homotypic targeting and self-recognition provides opportunities for personalized medicine. Despite their advantages as novel therapeutic agents, there are several challenges in optimizing cargo loading efficiency and structural stability and in defining exosome origins. Future research should include the development of large-scale, quality-controllable production methods, the refinement of drug loading strategies, and extensive in vivo studies and clinical trials. Despite the unresolved difficulties, the use of exosomes as efficient, stable, and safe therapeutic delivery systems is an interesting area in biomedical research. Therefore, this review describes exosomes and summarizes cutting-edge studies published in high-impact journals that have introduced novel or enhanced therapeutic effects using exosomes as a drug delivery system in the past 2 years. We provide an informative overview of the current state of exosome research, highlighting the unique properties and therapeutic applications of exosomes. We also emphasize challenges and future directions, underscoring the importance of addressing key issues in the field. With this review, we encourage researchers to further develop exosome-based drugs for clinical application, as such drugs may be among the most promising next-generation therapeutics.

## Exosomes: overcoming challenges for revolutionary biomedical applications

Nanomedicine, which applies nanotechnology in medicine, has improved drug delivery. Yet, issues like poor drug movement in the body, low absorption, and high toxicity of traditional drugs persist. Scientists are now using nanomedicines, especially nanoparticles, to tackle these problems. Nanoparticles can carry drugs to specific cells, help in imaging and diagnostic methods, or fix body structures at a cellular level. But, they can also cause unwanted immune responses. Hence, researchers are exploring exosomes, substances produced from various cells, as potential drug carriers. These naturally occurring exosomes are less toxic and more compatible with the body than synthetic nanoparticles. This study gives an overview of the current state of exosome research and its potential for drug delivery.

This summary was initially drafted using artificial intelligence, then revised and fact-checked by the author.

## Introduction

The use of many traditional drugs is often limited by their poor pharmacokinetics, low bioavailability, and high toxicity. Significant advances in overcoming these challenges and increasing the therapeutic efficacy of drugs have been made in the burgeoning fields of nanotechnology and nanomedicine. Nanomedicines represent a transformative and pervasive field of science in the 21st century, and nanomedicine is a specialized field that utilizes the knowledge and tools of nanotechnology for disease prevention and treatment. Nanomedicines typically include nanoparticles (NPs) that are 1 to 500 nanometers in size^1^. These tiny particles can be designed to deliver drugs to specific cells, facilitate imaging and diagnostic techniques, or even repair body structures at the cellular level. In addition, nanomedicines can be functionalized with drugs and targeted to specific sites. They offer advantages including high stability, tunable surface properties, and the possibility of combining therapeutic and diagnostic functionalities. For these reasons, nanomedicine is attracting significant research interest^2^.

NPs have attracted significant attention in the field of drug delivery due to their unique properties and ability to overcome various challenges associated with conventional drug delivery systems. NP-based DDSs (nanoparticle-based drug delivery systems) can be used to effectively transport therapeutic agents for the targeted treatment of diseases affecting various organ systems^3^. These DDSs include both organic and inorganic nanoparticles. Treatment agents can be encapsulated within organic nanoparticles, such as polymeric NPs and liposomes, to maximize their therapeutic effects. Surface modification with ligands can enable targeted drug delivery to specific cells or tissues. Like organic NPs, inorganic nanoparticles composed of materials such as gold, silver, iron oxide, and silica have also been utilized to load drugs and facilitate drug delivery for the treatment of diverse diseases. The use of NPs in DDSs can offer benefits such as increased drug solubility, prolonged release, targeted delivery, reduced toxicity, and heightened therapeutic efficacy^4^.

Although nanoparticles have great potential as drug carriers, adverse immunological responses to nanomedicines, including anaphylaxis, cytokine release syndrome, the neutralization of biological activity, cross-reactivity with endogenous protein counterparts, and delayed immune reactions, have led to termination of the production of various biologicals. Therefore, it is important to perform rigorous preclinical and clinical studies to examine factors including nanoparticle stability, biocompatibility, clearance mechanisms, and potential toxicity in order to ensure their safety and efficacy prior to their use in therapeutic applications^5,6^. Owing to the occurrence of adverse responses, many scientists have recently focused on exosomes, which are bioproducts generated from various cell lines and thus detected in various body fluids, including blood, urine, bronchoalveolar lavage fluid (BALF), and cerebrospinal fluid (CSF), as new potential drug carriers. Since they are naturally derived, exosomes typically exhibit lower toxicity and greater biocompatibility than synthetic NPs such as liposomes^7,8^. In addition, exosomes have a unique composition. They consist of a lipid bilayer membrane in which various membrane proteins, such as tetraspanins, glycoproteins, and signaling receptors, are embedded. This structure can naturally encapsulate various genetic substances, including DNAs, RNAs, and proteins. Accordingly, exosomes vary depending on their source and are affected by pathological conditions, which makes them candidate biomarkers for biopsy-based diagnostics^9^. In addition to their natural properties, exosome surface can be modified to expand the applications of exosomes in nanobiotechnology, particularly in drug delivery systems. The therapeutic application of exosomes depends on the cargoes they transport to target cells as well as on their own characteristics. Exogenous molecules with beneficial effects can be loaded into exosomes using various methods, such as electroporation, sonication, incubation, and/or transfection^10^.

Despite the current technical challenges related to exosome preparation and the efficiency of cargo loading, exosomes have promising therapeutic potential as a new type of drug delivery system. In this review article, we focused specifically on exosomes, which are the most extensively studied type of small extracellular vesicles^11^. We summarize basic information on exosomes, also known as small extracellular vesicles (sEVs); introduce cargo-loaded exosomes that are currently undergoing testing; and describe the therapeutic effects and advantages in exosome-based drug delivery. Finally, we discuss the therapeutic potential and future directions of this attractive field.

## Characteristics of exosomes

### Classification and biogenesis of exosomes

Extracellular vesicles (EVs) are highly heterogeneous membrane-bound carriers of various cargoes, including proteins, lipids, and nucleic acids. They are secreted by various cell types under both normal and pathological conditions, participate in intercellular communication, and influence various physiological and pathological processes. EVs are commonly classified as exosomes, microvesicles, or apoptotic bodies according to the mechanism of production^12^. The three types of EVs differ in their size, origin, and mechanism of formation, but they all serve as means of communication between cells. In addition, they carry various cargoes that reflect their cells of origin and can be delivered to other cells, influencing their function; thus, EVs are recognized as important biomarkers for diagnosis and therapy in many physiological and pathological conditions^13,14^.

**Fig. 1:**
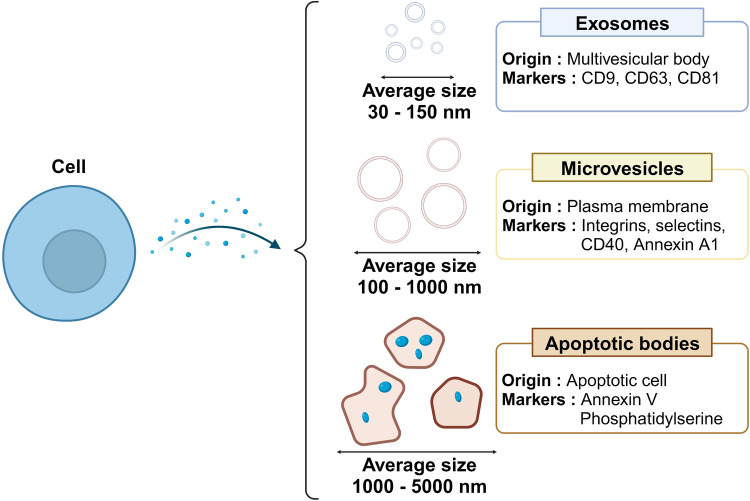
Classification of extracellular vesicles.

Exosomes are the smallest type of EV and are typically 30–150 nanometers in diameter. They originate from the endosomal system within cells. Exosomes contain a variety of molecular constituents, including proteins and RNA, from their cells of origin. Exosome can be taken up by neighboring or distant cells and influence their function. Microvesicles, also known as ectosomes, shedding vesicles, or microparticles, are larger than exosomes and have a diameter of ~100–1000 nanometers. They are formed by direct outward budding and fission of the plasma membrane. Like exosomes, microvesicles contain a variety of molecules from their cells of origin and can influence other cells by fusing with their membranes or by being internalized. Apoptotic bodies are the largest type of EV: they are usually 1–5 micrometers in diameter and are generated during apoptosis, a type of programmed cell death. The disassembling cell body forms blebs that then break off to form apoptotic bodies that contain various cellular components, including organelles and DNA. Apoptotic bodies are usually taken up and digested by nearby phagocytic cells and can influence the immune response (Fig. 1)^13^.

Although each type of EV exhibits distinct characteristics, separating exosomes and microvesicles can be challenging. Although exosomes are typically smaller, ranging from ~30 to 150 nm in diameter, and microvesicles are usually larger, from ~100 nm to 1 µm, there is a range in which they overlap, making them difficult to separate based solely on size. Although exosomes and microvesicles are generated through different cellular processes, there are no specific biomarkers that distinguish exosomes and microvesicles. Since both types of vesicles can contain similar proteins and RNAs, it is difficult to differentiate them based on their molecular content^15^. Therefore, the International Society for Extracellular Vesicles recommends classifying EVs into two main categories: small EVs (sEVs, with a diameter less than 200 nm) and medium/large EVs (m/lEVs, with a diameter greater than 200 nm)^16^. Since exosomes and sEVs are used interchangeably in research (the term “exosome” is preferred by researchers)^17^, we refer to exosomes and sEVs without distinguishing them in this review paper.

**Fig. 2:**
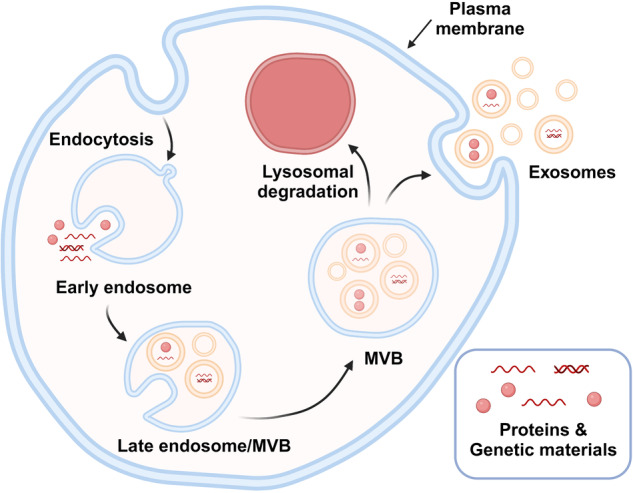
Biogenesis of exosomes.

The biogenesis of exosomes is a complex multistep process involving the endosomal pathway. It starts with the inward budding of the plasma membrane, a process called endocytosis. This process results in the formation of early endosomes, which subsequently mature into late endosomes. During maturation, late endosomes are formed by inward budding of the limited multivesicular body (MVB) membrane. The invagination of late endosomal membranes induces the formation of intraluminal vesicles (ILVs) within MVBs^18^. Throughout this process, proteins, nucleic acids, and lipids in the cytosol are incorporated into these small vesicles, which can fuse with the cellular membrane, leading to the release of ILVs as exosomes into the extracellular space. Alternatively, ILVs can merge with the lysosome if their contents are fated for degradation (Fig. 2)^19^.

### Cellular uptake of exosomes

**Fig. 3:**
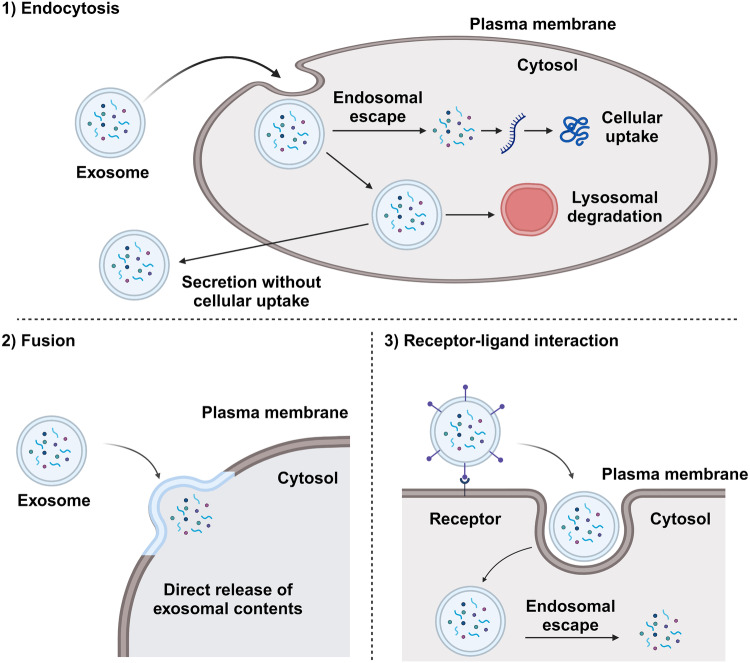
Cellular uptake pathways of exosomes.

Once exosomes are released into the extracellular space, they can interact with other cells in multiple ways. The specific mechanism may depend on factors such as the type of cells involved, the molecules present on the surface of the exosome, and the molecules present on the surface of the recipient cell. Currently, the mechanisms by which recipient cells can be targeted by exosomes are not fully understood, and whether the delivery of exosomes is largely random or specific to certain destinations remains unclear^20^. However, once an exosome reaches its recipient cells, it can be taken up via endocytosis, direct fusion with the plasma membrane, or interaction with cell-surface receptors, initiating intracellular signaling pathways (Fig. 3). Endocytosis is a process by which cells absorb material from outside their cell membrane by engulfing it with their cell membrane. Exosomes can be taken up by recipient cells through several forms of endocytosis, including clathrin-mediated endocytosis, caveolin-mediated endocytosis, macropinocytosis, and phagocytosis^21^. After endocytosis, exosomes can fuse with the endosome membrane to release their contents into the cytoplasm. Alternatively, exosomes can directly fuse with the plasma membrane of recipient cells, similar to how a viral envelope merges with the cell membrane during viral entry into a host cell^22^. This process releases the contents of the exosome directly into the cytoplasm of the recipient cell. Although endocytosis is the predominant mechanism for exosome uptake, this direct fusion route is the most effective for delivering cargo into the cell^23^. For exosomal molecular cargoes to exert effects within the recipient cell, they need to be released from vesicles into the cytoplasm, a process known as endosomal escape. However, if cargoes delivered via the endocytosis pathway remain within endosomes, they will be degraded by a lysosome or secreted into the extracellular space without any therapeutic effect on the target cell^24,25^. Thus, the direct fusion route of exosomes is more attractive for the use of exosomes as drug delivery vehicles. Once cargoes enter the cell, the contents of the exosome, such as proteins, lipids, RNA, and drugs, can influence the function of the recipient cell. These effects can include changes in gene expression, alterations in signaling pathways, and induction of immune responses. In the third route, specific proteins on the surface of exosomes can interact with specific receptors on the surface of recipient cells. Although the mechanism of interaction is unclear, exosome surface molecules, including fibronectin, tetraspanins, immunoglobulins, proteoglycans, lectin receptors, syncytin-1, and syncytin-2, are known to interact with target cells^26–28^. PD-L1, TNF, FasL, and TRAIL are currently the most therapeutically intriguing exosomal ligands due to their receptor presence on tumor cell surfaces, rendering them promising candidates for cancer treatment. However, the inconsistency of these procedures across diverse cell types is the main challenge in effectively applying exosome-based drug delivery systems in the clinic^29^. Nevertheless, many researchers are exploring the use of exosomes as next-generation drug delivery systems, primarily due to their ability to effectively deliver drugs into cells^30^.

### Drug loading methods

For several reasons, EVs have gained much attention in scientific research as next-generation carriers for drug delivery. Compared to synthetic nanoparticles, exosomes have shown lower toxicity and immunogenicity in preclinical studies. These findings suggested that exosomes may have a reduced risk of causing adverse reactions or immune responses when used as drug delivery vehicles. Exosomes are derived from cells and are formed by lipid bilayers, which are similar to cell membranes. This composition enhances the biocompatibility and stability of exosomes, allowing them to protect cargo molecules from degradation and harsh conditions in the body, such as enzymatic degradation and low-pH environments. Several researchers have reported that exosomes can reach their target organs after they travel in the bloodstream. Exosomes can even deliver drugs by penetrating the blood‒brain barrier (BBB)^31^. Therefore, many attempts have been made to load various therapeutic materials as well as naturally loaded substances, thereby increasing the therapeutic efficacy of these materials.

**Fig. 4:**
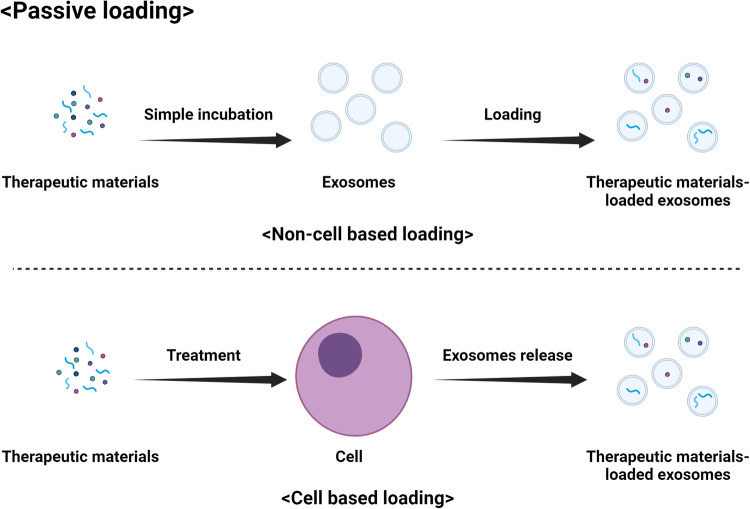
Passive loading of therapeutic materials into exosomes.

**Fig. 5:**
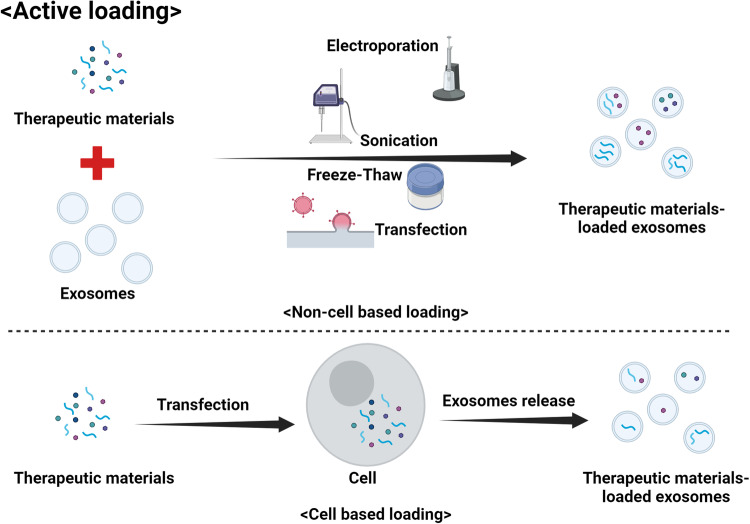
Active loading of therapeutic materials into exosomes.

Many ongoing studies have focused on optimizing the cargo loading of exosomes to utilize their full potential as drug delivery carriers. Various methods have been developed to load therapeutic agents, such as small-molecule drugs and genetic substances, into exosomes. There are two main methods used to load drugs into exosomes: passive loading (Fig. 4) and active loading (Fig. 5)^32^. The choice of method can depend on the specific drug and its properties, as well as the desired release mechanism. Passive loading occurs when cells are cultured with the drug of interest and naturally incorporate the drug into the exosomes as they form. The cells take up the drug, package it into exosomes, and then release the drug-loaded exosomes into the medium. This method is simple and easy to perform, but the loading efficiency is low, and it can be difficult to control the amount of drug loaded into the exosomes. Passive loading generally works best for lipophilic drugs that can easily cross cell and vesicle membranes. However, this method may not be appropriate for all drug types. Another method is active loading, in which the drug is directly loaded into isolated exosomes. This process is typically conducted by various techniques that increase the permeability of the exosome membrane, such as sonication, heat shock, electroporation, incubation with detergents, or the use of saponins. Active loading achieves higher loading efficiency than passive loading and allows more precise control of the drug loading amount. However, this approach can be complex and time-consuming, and there is a risk of damaging the exosomes if the process is not performed properly^10,32^. In both methods, removing the unloaded drug after loading by various purification techniques, such as ultracentrifugation, size exclusion chromatography, or ultrafiltration, is crucial. Genetic engineering can also be used to load genetic substances into exosomes. Cells can be genetically engineered to express a protein or RNA of interest, which can then be incorporated into exosomes as they form. This method can be used to load specific proteins or RNA molecules into exosomes but requires complex molecular biology techniques and may not be suitable for all types of therapeutic agents^10^. Each of these methods has advantages and disadvantages, and the best method can depend on factors such as the type of drug, the source of the exosomes, and the intended drug delivery target.

## Exosomes for therapeutic agent delivery

The interest in exosomes as drug carriers stems from several key attributes. Exosomes play a natural role in transporting various biological molecules between cells and have potential for use in the delivery of therapeutic agents^33^. In addition, exosomes are biocompatible and biodegradable, minimizing the risk of immune responses or toxicity that is often associated with synthetic drug delivery systems. The small size of these vesicles enables them to cross biological barriers that larger carriers cannot cross, and their stability in the bloodstream increases the chance of reaching their targets. Interestingly, exosomes have shown an inherent ability to target specific cells or tissues and can be utilized to deliver drugs directly to diseased cells, thereby increasing effectiveness and minimizing side effects^30^. Furthermore, exosomes are versatile and can carry cargoes including proteins, nucleic acids, and small drug molecules; thus, multiple therapeutic agents can be delivered simultaneously, and complex therapies involving various types of molecules can be designed^10^. This section focuses on the utilization of exosomes as a drug delivery system for improving therapeutic effects, categorizing studies based on the type of substances being delivered. The necessity of exosomes for the efficient delivery of each substance is discussed, and recent advances in research on exosomes as a vehicle for drug delivery are highlighted. Furthermore, the section highlights emerging strategies for modifying exosomes, including surface functionalization with targeting ligands or antibodies, to achieve targeted delivery to specific cells or tissues.

### Protein

Protein drugs, which include substances such as therapeutic recombinant proteins, monoclonal antibodies, and hormones, can interact with specific targets within the body to treat diseases or restore normal functions. However, they can lose their effectiveness through immune responses, clearance from the bloodstream, degradation by enzymes, or binding to other molecules. To mitigate these challenges, carriers such as nanoparticles and exosomes can be used^34^. Carriers can increase the stability and bioavailability of these drugs, control their release for sustained effects, enable targeted delivery, facilitate cell penetration, promote elevated therapeutic effects, and prevent immune responses^35^. Thus, carriers play an indispensable role in improving the effectiveness and efficiency of protein delivery (Table 1).

**Table 1:** Loading protein cargo into exosomes.

Exosome origin	Protein cargo	Loading method	Application	Reference
Rat platelet	Yap1	Direct electroporation into exosomes	Rejuvenation of tendon	Lu et al.^37^
Breast cancer cells (MCF-7, SK-BR-3, and 4T1)	P53	Direct electroporation into exosomes	Breast cancer	Jiao et al.^40^
Mouse serum	DNase I	Copper-free click chemistry	ARDS (neutrophil extracellular trap [NET] inhibition and M2 polarization)	Liu et al.^41^
Lung epithelial cell (BEAS-2B)	CC16	Donor cell overexpression	Acute lung injury	Han et al.^43^
Mesenchymal stem cells	IL-27	Donor cell overexpression	Inflammatory bowel disease	Nie et al.^44^
Liver-circulated fluids	TM4SF5	Generation of overexpression mice	Type 2 diabetes	Jung et al.^45^
Primary dermal fibroblasts from BALB/c mouse	IL-4, IL-10	Donor cell overexpression	Acute lung injury	Salazar-Puerta et al.^46^
HEK293T	Wnt3a	Donor cell overexpression	Alveolar epithelial regeneration	Gao et al.^47^
HEK293T	Ovalbumin with CpG-DNA	Donor cell overexpression	Allergic rhinitis	Liu et al.^48^
Mesenchymal stem cell	PD-L1	Priming donor cells with TNF-α and IFN-γ	Type 1 diabetes	Wang et al.^49^
HK-2	TNFAIP8	Priming donor cells with TGF-β	Kidney fibrosis	Liu et al.^50^
Mesenchymal stem cell	Brain-derived neurotrophic factor (BDNF)	Donor cell overexpression	Ischemic stroke	Zhou et al.^51^
Immature dendritic cells	AMPKα1 dominant negative mutant	Direct transfection into exosomes	Genetic obesity	Milbank et al.^52^

In some studies, researchers have attempted to load therapeutic proteins into exosomes indirectly. Misfolded proteins were encapsulated in exosomes by treatment with bafilomycin A1, which can produce many unfolded proteins by impairing lysosomal function^36^. Therefore, large numbers of misfolded proteins were loaded into exosomes, leading to cell death upon delivery to cancer cells. However, it is difficult to determine which proteins can be loaded, and the proteins being loaded cannot be precisely controlled; thus, there have also been attempts to load desired proteins into exosomes directly. Lu et al. loaded recombinant Yap1 into rat platelet-derived exosomes directly through electroporation^37^. Yap1-loaded exosomes promote the rejuvenation of tendon stem/progenitor cells for functional tendon regeneration. However, soluble proteins, which have a hydrophilic outer surface with a hydrophobic core, cannot easily penetrate the lipid bilayer structure of exosomes^38,39^. To increase the efficiency of protein loading into exosomes, Jiao et al. conducted electroporation to empty the exosome of its original contents, thereby maximizing the efficiency of loading exogenous recombinant P53 into exosomes^40^. There have also been attempts to use proteins to precisely deliver drugs to target cells or tissues. Liu et al. conjugated DNase I to exosome and liposome hybrid particles loaded with methylprednisolone sodium succinate (MPS), an ARDS treatment drug, through copper-free click chemistry. By this method, MPS was effectively delivered to macrophages, where it exerted an enhanced therapeutic effect^41^.

Although the direct loading of proteins into exosomes is promising, the exposure of the protein surface in solution and the low refolding rate in target cells, which can decrease protein stability and activity, are important obstacles to the delivery of recombinant proteins^42^. In addition, complex modification processes are often necessary to increase the loading efficiency of proteins into exosomes. As a result, many researchers are trying a different strategy in which donor cells are transfected to overexpress specific therapeutic proteins. This approach allows overexpressed proteins to be naturally incorporated into exosomes, significantly simplifying the process. Han et al. overexpressed CC16 in BEAS-2B lung epithelial cells and then isolated exosomes from the culture medium^43^. In particular, CC16-loaded exosomes exerted a protective effect against lung injury at concentrations more than 1000 times lower than that required for recombinant CC16 to obtain a similar effect. Nie et al. transfected mesenchymal stem cells (MSCs) with lentivirus to overexpress IL-27 and thereby generated IL-27-loaded exosomes. In this study, IL-27-loaded exosomes inhibited inflammatory responses and rescued intestinal barrier dysfunction^44^. Jung et al. generated hepatic TM4SF5-overexpressing (Alb-Tm4sf5 TG) mice and isolated TM4SF5-loaded exosomes from liver-circulating fluids. The administration of TM4SF5-loaded exosomes to TM4SF5 knockout mice induced the clearance of excess glucose clearance by targeting brown adipose tissues^45^. Several researchers have transfected several plasmids together to increase the therapeutic efficacy. Salazar-Puerta et al. transfected IL-4 or IL-10 together with an SPA ligand, which can increase intrapulmonary/alveolar interactions and retention, into primary fibroblasts and isolated exosomes (IL-4 + SPA exo or IL-10 + SPA exo) that had therapeutic effects in acute lung injury^46^. Gao et al. transfected Wnt3a together with engineered glypican GPC6^ΔGPI^‐C1C2, which is a stabilizer anchor, and isolated exosomes from the resulting Wnt3a-GPC6^ΔGPI^‐C1C2 cells that promoted epithelial regeneration more effectively than exosomes isolated from cells transfected with Wnt3a alone^47^. Liu et al. regulated the Th1 immune response by treating cells with CpG DNA and ovalbumin-loaded exosomes, alleviating allergic symptoms^48^. Wang et al. attempted to increase the therapeutic efficacy of the PD-L1 protein by loading an additional FDA-approved drug^49^. Mesenchymal stem cells (MSCs) were primed with TNF-α and IFN-γ for 48 h to obtain PD-L1-loaded exosomes. Then, hexyl 5-aminolevulinate hydrochloride (HAL) was loaded into PD-L1-loaded exosomes by electroporation and used to treat type 1 diabetes by preventing the apoptosis of islet β cells. Unlike other studies, in this study, specific proteins were indirectly loaded by stimulating donor cells. In this indirect method, various unexpected proteins can be loaded in addition to the intended protein. This result was also confirmed in a study by Liu et al.^50^. Exosomes were isolated from TGF-β-primed tubular epithelial cells (HK-2 cells). Tumor necrosis factor-α-induced protein 8 (TNFAIP8)-loaded exosomes were found to be enriched in TGF-β-primed HK-2 cell culture medium, and these exosomes effectively prevented kidney fibrosis. In this study, TNFAIP8 was not the only protein loaded by the indirect method used. For this reason, the exosome cargo in addition to the intended proteins should be identified. Additionally, any effects arising from exosomes derived from unintended loaded proteins should be confirmed. The indirect loading of specific proteins should be reviewed carefully to ensure the precise contents and effects of the exosomes.

Exosomes can naturally cross the BBB and enter the brain, making them attractive candidates for drug delivery to treat various neurological disorders. Zhou et al. overexpressed brain-derived neurotrophic factor (BDNF) in MSCs to purify BDNF-loaded exosomes. BDNF-loaded exosomes were then administered to mice via intranasal delivery and shown to affect ischemic stroke by penetrating the BBB^51^. Although exosomes can delivery drugs into the brain by penetrating the BBB, their delivery efficiency is still very low. Therefore, researchers have tried to increase delivery efficiency by engineering exosomes. Milbank et al. used immature dendritic cells overexpressing lysosome-associated membrane protein 2b (Lamp2b) and neurotrophic rabies virus (RVG), which enables more efficient penetration of the BBB by binding to the nicotinic acetylcholine receptor (nAChR). After exosomes were isolated from engineered immature dendritic cells, AMPKα1 dominant negative mutant plasmid-transfected exosomes were delivered to the ventromedial nucleus of the hypothalamus, promoting weight loss in leptin receptor-deficient mice^52^.

### Genetic materials

Genetic materials, such as small interfering RNA (siRNA), microRNA (miRNA), messenger RNA (mRNA), and DNA, have been used extensively in gene therapy. In particular, siRNAs, miRNAs, and antisense oligonucleotides (ASOs) are commonly used in function loss research. However, cells have a protective lipid bilayer membrane that acts as a barrier to the direct entry of large and hydrophilic molecules such as siRNAs, miRNAs, and ASOs. These genetic materials are negatively charged and cannot easily pass through the cell membrane, hindering their access to the intracellular machinery responsible for gene regulation^53^. They are also susceptible to enzymatic degradation, which hinders their effective transportation to cells; thus, they require a carrier or delivery system for effective transport into cells (Table 2).

**Table 2:** Loading genetic materials into exosomes.

Exosome origin	Loaded material	Loading method	Application	Reference
C57BL/6J mice plasma	Anti-H19 (small RNA)	Injection of anti-H19 into mice via tail vein	Colorectal cancer	Sun et al.^56^
Adipose-derived mesenchymal stem cells	NF-κB siRNA	Donor cell transfection	Skin injury	Lu et al.^57^
Neural stem cells (iNSCs)	CCL2-siRNA	Direct electroporation into exosomes	Traumatic spinal cord injury	Rong et al.^58^
Mouse serum	MyD88-siRNA	Direct electroporation or CaCl_2_ transfection into exosomes	Acute lung injury	Han et al.^61^
Mesothelial cells	miR-769-5p	Donor cell transfection	Organ fibrosis	Bontempi et al.^62^
Ckit+ progenitor cells	miR-126	Direct electroporation into exosomes	Myocardial infarction	Bheri et al.^63^
Neural stem cells	STAT3 ASO	Donor cell treatment	Glioma	Adamus et al.^67^
HEK293	GSDMD-N mRNA	Donor cell transfection with puromycin treatment	Breast tumor	Xing et al.^68^
HEK293T	LDL-R mRNA	Donor cell transfection	Familial hypercholesterolemia	Mei et al.^69^, Yang et al.^70^
Neonatal human dermal fibroblasts	COL1A1 mRNA	Donor cell transfection	Photoaged skin	You et al.^72^
HEK293T cells	i*Bax* mRNA	Donor cell transfection	Atherosclerosis by targeting senescent cells	Zhang et al.^73^
HEK293T	ALKBH5 mRNA	Fusion with ALKBH5 mRNA-loaded liposome	Colorectal cancer	Wu et al.^74^
Mesenchymal stem cell	A151 oligodeoxynucleotide	Direct incubation with exosome	Immunomodulation and skin regeneration	Camoes et al.^75^
HEK293T	RBP-J decoy oligodeoxynucleotides	Direct electroporation into exosomes	Hepatic fibrosis	He et al.^76^
Osteosarcoma cells	MEG3 (long noncoding RNA)	Donor cell transfection	Osteosarcoma	Huang et al.^77^
Mesenchymal stem/stromal cells	HOTAIR (long noncoding RNA)	Donor cell transfection	Angiogenesis and wound healing	Born et al.^78^
HEK293T	mSCAR (circular RNA)	Donor cell transfection	Sepsis	Fan et al.^79^
HEK293T	AAV9-SERCA2a	Donor cell transfection	Myocardial infarction	Li et al.^80^
HEK293FT	Cas9–single-guide-RNA complexes for C-X-C chemokine coreceptor type 4	Donor cell transfection	HIV-1	Stranford et al.^83^

Several limitations and challenges have hindered the widespread clinical use of these materials. Although various carriers, including polymers, cyclodextrins, and liposomes, are used in clinical applications due to their high transfection efficiency in vitro, the in vivo performance of most delivery systems is unsatisfactory due to toxicity, nonspecific uptake, and unwanted inflammatory and immune responses^54,55^. Various delivery systems present problems such as off-target effects, limited tissue penetration, and rapid clearance, which can reduce therapeutic efficacy and cause unintended side effects. Therefore, targeted delivery of siRNAs, miRNAs, or ASOs to specific tissues or cells in the body remains a significant challenge. In efforts to overcome these limitations, exosomes have attracted significant attention as potential carriers for gene therapy. Sun et al. constructed a small RNA, anti-H19, that targets H19 lncRNA. They confirmed that overexpressed anti-H19 could be loaded into exosomes. They loaded anti-H19 into mouse plasma exosomes by injection These exosomes ameliorated colorectal cancer in a nontoxic, nonimmunogenic, and biocompatible manner. Their therapeutic effect was better than that of 5-Fu, one of the most commonly used cancer drugs^56^. Lu et al. loaded NF-κB siRNA into adipose-derived mesenchymal stem cell exosomes and treated skin injury by inhibiting NF-κB inflammatory signaling^57^. Since exosomes are natural extracellular vesicles secreted by many cell types, they can offer better biocompatibility and reduced immunogenicity than synthetic nanoparticles. In addition, exosomes can be engineered to carry specific target molecules on their surface, allowing them to interact selectively with specific cell types and to be delivered intracellularly with high efficiency, thereby reducing off-target effects, which commonly induce side effects. CAQK peptide selectively binds chondroitin sulfate proteoglycans (CSPGs), whose expression is upregulated in lesions after spinal cord injury, was anchored to the membranes of neural stem cell (iNSC)-derived exosomes through chemical modification in a study by Rong et al. The exosomes were then loaded with CCL2-siRNA for delivery to the spinal cord to treat injury^58^. In addition to targeting functions, siRNA-loaded exosomes can be delivered through medical devices such as nebulizers. It has been reported that nebulizers can affect drug stability by impacting molecular integrity, leading to a drug loss of up to 88% during the initial loading into liposomes^59,60^. However, there was no difference in the amount of siRNA loaded into exosomes before and after nebulization, and the loaded siMyD88 inhibited the NF inflammatory pathway by inhibiting NF-κB inflammatory signaling^61^. Currently, progress in the development of siRNAs is outpacing that of miRNAs, with a greater number of siRNA candidates already in clinical trials. This trend can be attributed to the complex roles and multiple targets of miRNAs. However, there has been a recent increase in intensive research focusing on miRNAs, and it is anticipated that significant advances will be made in their therapeutic potential^61^. Bontempi et al. loaded miR-769-5p into mesothelial cell-derived exosomes, inhibiting organ fibrosis by blocking the mesothelial-to-mesenchymal transition^62^. Bheri et al. loaded miR-126 into cKit+ progenitor cells to ameliorate myocardial infarction^63^. In addition, attempts to maximize the delivery efficiency of miRNAs have been ongoing. Modifying exosomes by hybridizing them with liposomes, hydrogels, or peptides can increase their stability and targeting ability^64–66^. Similar to siRNA and miRNA, ASOs, which are small synthetic fragments of nucleic acids block the function of proteins by binding to mRNAs, which can lead to mRNA degradation, thereby altering protein production. To deliver ASOs into cells, carriers are needed. In the research of Adamus et al., glioma-targeted STAT3-ASOs were shown to function only when they were delivered through carriers such as exosomes^67^.

Moreover, mRNAs can be loaded into exosomes. Xing et al. loaded GSDMD-N mRNA, one of the key factors involved in pyroptosis. Briefly, they transfected the GSDMD-N plasmid into HEK293 cells with puromycin treatment. Since puromycin inhibits translation, untranslated GSDMD-N mRNA can be easily sorted into exosomes. Through this process, they successfully loaded GSDMD-N mRNA into exosomes^68^. However, loading mRNA into nanocarriers is still challenging due to the instability and large size of the mRNA, which complicates packaging and controlled release. Even after successful loading and sorting, controlled release of mRNA at the target site is challenging, as rapid release may result in degradation before the desired effect is achieved. Therefore, Yang et al. developed an “All-in-One” exosome engineering and purification strategy for easy packaging. Briefly, they generated HEK-293T cells expressing Flag-TCS-PTGFRN-CTSL-MCP, namely, an exosome sorter and the mRNA Ldlr-MS2. Since MCP can specifically bind to MS2-containing RNA during biogenesis, the therapeutic mRNA Ldlr-MS2 was loaded into exosomes conjugated with Flag-TCS-PTGFRN-CTSL-MCP. Then, the exosomes were purified with anti-Flag magnetic beads. After purification, Flag and MCP were cleaved by thrombin treatment and acidification, respectively^69^. To release Ldlr mRNA more efficiently, they delivered the Ldlr releaser, which can release Ldlr mRNA from MS2 by competitively interacting with MS2^70^. Through this process, therapeutic Ldlr mRNA can be delivered into cells to exert a therapeutic effect on atherosclerosis. Despite the complex loading process compared to that of other materials, advancements are being made, highlighted by the successful use of lipid nanoparticles for mRNA delivery in COVID-19 vaccines. Currently, many efforts are underway to hybridize exosomes and load them with sufficient amounts of mRNA for clinical use. Several research groups have attempted to hybridize exosomes with other engineering technologies. Yang et al. applied cellular nanoporation by using a biochip that generates electrical pulses that stimulate cells to release additional exosomes with increased mRNA loading^71^. Through this technology, You et al. loaded COL1A1 mRNA into exosomes and prevented wrinkle formation during photoaging of the skin^72^. Various studies have involved attempts to increase the efficacy of mRNA function. Zhang et al. generated the i*Bax* mRNA, a modified *Bax* mRNA that targets the liver-specific miRNA miR-122, to modulate hepatotoxicity caused by excessive translation of i*Bax*. Then, this mRNA was encapsulated into exosomes conjugated with superparamagnetic iron oxide nanoparticles (SMNs). This approach enabled the use of magnetic fields to target lesions precisely to ameliorate atherosclerosis^73^. In addition, exosomes and liposomes can be used to form hybrid nanoparticles to increase loading efficiency while maintaining low toxicity. Wu et al. constructed exosome–liposome hybrid nanoparticles through the freeze−thaw method. A mixture of exosomes and ALKBH5 mRNA-loaded liposomes (1:1) was repeatedly frozen and thawed three times to induce fusion of the two particles. This process can solve the difficulty of encapsulating mRNA in exosomes and ameliorate the toxicity of liposomes, resulting in a greatly improved effect on colorectal cancer^74^. In addition to these genetic materials, researchers have successfully loaded various genetic materials into exosomes, including oligodeoxynucleotides^75,76^, long noncoding RNAs^77,78^, circular RNAs^79^, and adenoviruses^80^. In particular, clustered regularly interspaced short palindromic repeats (CRISPR), which of particular interest in genetic therapy, is also being studied intensively for loading into exosomes for targeted delivery^81,82^. Stranford et al. improved the exosome-based CRISPR gene delivery system by generating genetically programmed self-assembling multifunctional particles. This system avoided multiple challenges, including purification and the decreased exosome yields resulting from the chemical modification of sgRNA^83^. At the end of 2023, CRISPR gene therapy for sickle cell disease received initial approval from the FDA, and its use is expected to greatly increase in the future^84^.

### Delivery of small-molecule drugs

Natural vesicle-derived exosomes offer advantages including a decreased immune response, reduced toxicity, enhanced bioavailability, and potential for selective drug delivery. Therefore, exosomes can reduce the drug doses needed to achieve the same therapeutic effects and decrease side effects (Table 3). Reddy et al. loaded bevacizumab into exosomes and observed the resulting therapeutic effects on diabetic retinopathy for 2 months. Bevacizumab alone exhibited observable effects for 1 month. In contrast, bevacizumab-loaded exosomes exhibited effects for 2 months, demonstrating the ability of the exosome drug delivery system to prolong the effectiveness of a drug^85^. Zhou et al. loaded the caspase-1 inhibitor VX-765 into bone marrow dendritic cell-derived exosomes. In this study, the therapeutic efficacy of VX-765 was increased by exosomal delivery to macrophages^86^. Exosomes are also promising for overcoming drug resistance and can be modified to achieve targeted delivery and high loading efficiency. Iyaswamy et al. modified the surface of exosomes to express the amyloid-β precursor protein (APP)-binding protein Fe65. Then, corynoxine-B was loaded into exosomes to induce autophagy in APP-expressing neuronal cells, and treatment with these exosomes ameliorated cognitive decline and pathogenesis in a mouse model of Alzheimer’s disease^87^. Due to these advantages, many researchers are investigating the potential of loading exosomes with anticancer drugs to address the shortcomings of conventional anticancer drugs, including poor bioavailability, the necessity of high doses as a result of nonspecific targeting, low therapeutic indices, and multiple drug resistance^88,89^. Using exosomes, Rehman et al. maximized the anticancer effect of temozolomide, a brain-specific cancer drug, on temozolomide-resistant glioblastoma^90^. To target glioblastoma, they decorated exosomes with the short peptide HSSP. HSSP can specifically bind to heme oxygenase-1 (HMOX1), which is highly expressed in temozolomide-resistant glioblastoma. These exosomes were loaded with a combination of temozolomide and siSTAT3, which can inhibit the cancer progression gene STAT3. These HSSP-decorated exosomes carrying temozolomide and siSTAT3 reached the brain in larger quantities than other experimental groups and exhibited the strongest anticancer effects. Zhu et al. modified the surface of exosomes to more effectively target the brain. Angiopep-2 targets LRP1, which is highly expressed in glioblastoma cells, and TAT peptide is a cell-penetrating peptide. Both molecules were overexpressed in HEK293T cells, and angiopep-2/TAT-loaded exosomes were isolated. Doxorubicin, a chemotherapeutic agent for treating various cancers, was directly loaded into these Angiopep-2/TAT-loaded exosomes via electroporation, and survival time was significantly improved in a mouse model of glioma without severe side effects^91^. To increase the efficacy of cancer immunotherapy, Kim et al. loaded doxorubicin into signal regulatory protein alpha (SIPRα)-loaded exosomes. Since SIPRα can block CD47, a tumor antigen related to cancer immunotherapy, SIPRα-loaded exosomes can target cancer cells for immunotherapy, and the loaded doxorubicin increases the anticancer effect^92^. Faruque et al. isolated exosomes from human pancreatic cancer cells and loaded them with RGD peptides to bind to αvβ3, which is highly expressed in pancreatic cancer cells. These pancreatic cancer cell-targeting exosomes were then loaded with paclitaxel and were confirmed to have advantageous anticancer effects^93^.

**Table 3:** Loading small molecules into exosomes.

Exosome origin	Loaded materials	Loading method	Application	Reference
Mesenchymal stem cell	Bevacizumab	Direct incubation with exosomes	Diabetic retinopathy	Reddy et al.^85^
Bone marrow dendritic cells	VX-765	Direct sonication with exosomes	Myasthenia gravis	Zhou et al.^86^
OVCAR-8 cells	Benzoyloxy dibenzyl carbonate (B2C)	Direct sonication with exosomes	Multidrug-resistant cancer	Kang et al.^89^
HT22 hippocampus neurons	Corynoxine-B	Direct sonication with exosomes	Alzheimer’s disease	Iyaswamy et al.^87^
Bone marrow mesenchymal stem cells	Temozolomide with siSTAT3	Direct sonication with exosomes	Temozolomide-resistant glioblastoma	Rehman et al.^90^
HEK293T	Doxorubicin	Direct electroporation into exosomes	Glioma	Zhu et al.^91^
HEK293T	Doxorubicin	Direct incubation with exosomes	Melanoma	Kim et al.^92^
Human pancreatic cancer cell (PANC-1)	Paclitaxel	Direct sonication with exosomes	Pancreatic cancer	Al Faruque et al.^93^
RAW264.7	Shikonin, photosensitizer IR820, and polymetformin	Fusion with shikonin-, photosensitizer IR820-, and polymetformin-loaded liposomes	Breast cancer and melanoma under laser irradiation	Tang et al.^94^
Goat milk	Chlorin e6 and 18F-FDG	Direct incubation with exosomes	Breast cancer	Guo et al.^95^
HEK-293T	FX11 (a glycolysis inhibitor)	Direct incubation with exosomes	Breast cancer	Nguyen Cao et al.^96^
Brain endothelial cells (bEnd.3 cells	Ce6	Direct incubation with exosomes	Brain cancer	Nguyen Cao et al.^97^
HEK293T	Triphenylphosphonium (TPP) conjugated chlorin e6 and piperlongumine	Direct incubation with exosomes	Breast cancer	Nguyen Cao et al.^98^
HEK293T	Indocyanine green paclitaxel, and sodium bicarbonate	Direct incubation with exosomes	Breast cancer	Nguyen Cao et al.^99^
4T1 or CT26	GM-CSF mRNA	Donor cell transfection	Colon and breast cancers	Ji et al.^100^
RAW264.7	Resveratrol	Direct sonication with exosomes	Multiple sclerosis	Zheng et al.^102^
Milk	Forsythiaside A	Direct ultrasonic incubation with exosomes	Liver fibrosis	Gong et al.^103^
J774A.1	Curcumin and albumin	Direct sonication with exosomes	Inflammatory skin diseases	Yerneni et al.^104^

In addition to exosome surface modification, several engineering technologies have been applied to exosomes. Tang et al. inserted the CD47-targeting RS17 peptide into liposomes with the chemotherapeutic agent shikonin, the photosensitizer IR820, and the immunomodulator poly-metformin. These liposomes were then fused with exosomes and used to treat breast cancer and melanoma under laser irradiation^94^. Several researchers have applied chlorin e6 (Ce6), which is an FDA-approved photosensitizer. Photodynamic therapy (PDT) is a two-stage cancer treatment method that utilizes light energy combined with a photosensitizer that can be activated by light energy. Guo et al. loaded Ce6 with 18F-FDG, which can exert photodynamic effects against cancer cells, into goat milk-derived exosomes and thereby targeted breast cancer for Cerenkov luminescence-induced photodynamic therapy^95^. Nguyen Cao et al. also loaded Ce6 into exosomes for sonodynamic therapy, which is a novel ultrasound-based noninvasive therapeutic strategy derived from photodynamic therapy^96^. Briefly, Ce6 was conjugated with a triphenylphosphonium (TPP) moiety to target mitochondria. This conjugate was subsequently loaded into brain endothelial cell-derived exosomes to treat brain cancer^97^. They also utilized TPP-Ce6-engineered exosomes to load the cancer-specific chemotherapeutic agent pipelongumin (PL). After ultrasonic irradiation, TPP-Ce6-PL-loaded exosomes exerted the strongest anticancer effect on breast cancer^98^. This research team also coloaded indocyanine green, a sonosensitizer and photoacoustic agent, with paclitaxel and sodium bicarbonate and confirmed the anticancer effect on breast cancer^99^. Ji et al. also utilized Ce6 with CCL21a and GM-CSF mRNA to treat colon and breast cancers^100^.

Natural product-derived chemicals have also been loaded into exosomes to increase their therapeutic efficacy. Natural product-derived chemicals often have lower potency than synthetic drugs^101^. However, natural product-derived chemicals loaded into exosomes exhibited much higher therapeutic efficacy than the natural product-derived chemicals alone. For example, resveratrol-loaded exosomes showed increased anti-inflammatory effects compared to resveratrol alone^102^. Gong et al. loaded *Forsythiae Fructus*-derived forsythiaside A into hyaluronic acid-modified exosomes; because hyaluronic acid can bind to CD44, which is overexpressed in fibrotic tissue, the forsythiaside A-loaded exosomes were targeted to fibrotic sites and showed the ability to regulate NLRP3-mediated pyroptosis^103^. In addition, curcumin-loaded exosomes have been administered via dissolvable microneedle arrays, further expanding the use of natural products by improving their therapeutic function^104^. Numerous similar studies on delivering natural products combined with genetic materials are in progress, and high-impact research similar to that mentioned in this review is steadily being published. Thus, exosome-based approaches are expected to introduce a new chapter in natural product research.

## Conclusion and future directions

Because of their biocompatibility, deep tissue penetration, and versatile cargo-loading capacity, exosomes are emerging as promising carriers for targeted drug delivery. Owing to their homotypic targeting effects and self-recognition capabilities, exosomes and their hybrid systems are being increasingly explored as alternatives to conventional drug carriers^31^. These materials have potential for various applications, including chemotherapy, gene therapy, and photothermal therapy^30^. In addition, multifunctional exosomes can be generated by the simultaneously loading of various therapeutic cargoes^94,105,106^. Moreover, while mammalian-derived exosomes have been extensively researched in recent years, increasing evidence also indicates that plant-derived exosome-like nanovesicles exhibit structural and functional similarities to mammalian-derived exosomes^107^, suggesting that the potential of utilizing exosomes is still far from fully understood. However, exosome research and development still present significant challenges related to loading efficiency, structural stability, and defining exosome origins. The existing strategies for cargo loading, such as incubation, transfection, electroporation, sonication, and in situ synthesis, present limitations in terms of efficiency, cost, potential exosome damage, and versatility of the loaded cargo^108^. Furthermore, substantial losses can occur during the preparation of modified exosomes or the encapsulation of drugs, leading to low yield and poor reproducibility. To optimize the use of exosomes for targeted delivery, various strategies for assembling homing molecules on exosomal surfaces have been developed. Biological strategies leveraging ligand–receptor interactions offer high specificity but demand broader identification of cell-, tissue-, and organ-specific receptors^58,92–94^. Additionally, clinical application requires continued research, larger animal studies, clinical trials, and intensive efforts to achieve large-scale, quality-controllable production. Despite these challenges, the unique attributes of exosomes, including high targeting ability, nontoxicity, and biocompatibility, underscore their substantial potential for drug delivery with broad public health benefits^5,6^. Therefore, continued exploration and innovation in developing efficient, stable, and safe exosome-based delivery systems are encouraged.

First, a more comprehensive understanding of exosome biology is needed. For instance, detailed studies to uncover the precise mechanisms governing the biogenesis, cargo sorting, release, and uptake of exosomes could be paramount in deciphering the roles of exosomes in intercellular communication and pathological conditions, such as cancer metastasis and neurodegenerative diseases^109^. Overexpressing specific proteins and genes in exosomes may unexpectedly result in the expression of subdomain proteins. Therefore, understanding how defined stimuli affect the sorting of proteins into modified exosomes is highly important for elucidating therapeutic mechanisms in more detail^110^. Second, increasing the loading efficiency and stability of exosomes is crucial. Current techniques such as electroporation, sonication, or incubation with permeabilization agents have shown potential but also limitations, such as effects on exosome integrity^10^. Further investigations of these techniques or the development of novel methods will play important roles in optimizing exosome-based therapeutic delivery. Third, the scalability of exosome production and purification poses a formidable challenge^30^. Currently, exosomes are typically isolated from cell culture media or bodily fluids through ultracentrifugation, ultrafiltration, or size exclusion chromatography, methods that often suffer from low yield or purity. Developing techniques that can efficiently yield high-quality exosomes on a larger scale is important for future clinical application^111^. Zhang et al.^112^ developed cationic lipid‒polymer hybrid nanoparticles that encapsulate a cascade system involving catalytic hairpin assembly and CRISPR–Cas12a (CLHN–CCC), allowing exosome enrichment in a three-dimensional space. The enrichment of exosomes via this method is advantageous for miRNA detection and early-stage cancer diagnosis^113^. Furthermore, the translation of exosome research into clinical practice necessitates successful clinical trials. Several exosome-based products, such as ExoFlo for acute respiratory distress syndrome and dexosomes for melanoma treatment, are in various stages of clinical trials. The continuation of these trials and the initiation of new ones will be crucial for assessing the safety, efficacy, and optimal dosage of exosome-based therapeutics. In a study by Meng et al.^114^, the intradermal maintenance of exosomes was extended for more than twice as long (up to 7 days) by delivering the exosomes through micro-subcutaneous injection via a dissolvable microneedle. This approach enabled exosomes to effectively ameliorate skin damage. However, the preparation and storage lifetime of microneedles have not yet been fully elucidated. Therefore, techniques for exosome storage, like those available for liposomes, should be developed to address the inconvenience of exosome storage^115^. Finally, the potential off-target effects and long-term safety of exosome-based therapies must be thoroughly investigated^116^. For instance, exosomes derived from tumor cells have been shown to potentially promote tumor growth or metastasis, which necessitates a cautious approach to their therapeutic application^117^. In addition, the development of standardized protocols for exosome research is vital for future progress. Currently, the lack of uniformity in exosome isolation, characterization, and storage techniques often hampers the comparison of results across studies. Efforts to establish consensus and standardize these procedures will benefit the research community. In conclusion, while exosome research holds immense potential, the path forward must address these important challenges and opportunities to fully realize the transformative possibilities of this field.
